# The Transcription Factor Otc4A Stimulates the Proliferation, Invasion, and Stemness of Colorectal Cancer Cells by Inhibiting the Regulation of miR-7-5p on TLR4

**DOI:** 10.1155/2022/7856629

**Published:** 2022-09-26

**Authors:** Jinsong He, Liang Duan, Yu Xie, Shoujiang Wei

**Affiliations:** ^1^Department of Gastroenterology, Affiliated Hospital of North Sichuan Medical College, Nanchong, Sichuan 637000, China; ^2^Department of Gastroenterology, Quxian People's Hospital, Dazhou, Sichuan 635299, China

## Abstract

**Background:**

To investigate the effects and mechanism of octamer-binding transcription factor 4 (Otc4A) on proliferation, invasion, and stemness of colorectal cancer (CRC) cells.

**Methods:**

Firstly, normal fetal human cells (FHC, colon epithelial cells) and HT29 cells (CRC cells) were cultured. The expression levels of Otc4A, miR-7-5p, and TLR4 in cells were then detected by qRT-PCR. CCK-8 was adopted to measure cell proliferation rate after Otc4A, miR-7-5p, and TLR4, respectively, were either knocked out or overexpressed in HT29 cells. Later, the cell viability was detected by cell cloning assay; cell invasion by transwell; cell sphere-forming ability by sphere-formation assay; protein expression level of Otc4A, p65, p-p65, and TLR4 by western blot; and the targeting relationships between miR-7-5p and Otc4A as well as miR-7-5p and TLR4 by dual-luciferase reporter assay. Finally, chromatin immunoprecipitation was applied to verify the interaction between Otc4A and miR-7-5p.

**Results:**

In HT29 cells, Otc4A expression was significantly increased. Additionally, the knockdown of Otc4A prevented HT29 cells from proliferating, migrating, forming spheres, and activating NF–B signaling. Otc4A could negatively regulate miR-7-5p, and miR-7-5p could target TLR4 expression. Besides, a negative correlation was found between Otc4A and miR-7-5p. Finally, the knockdown of miR-7-5p or overexpression of TLR4 could significantly reverse the effect of the knockdown of Otc4A on HT29 cells.

**Conclusion:**

The transcription factor Otc4A can regulate the level of TLR4 by inhibiting the expression of miR-7-5p and then promote the proliferation and invasion of CRC cell HT29 as well as enhance cell stemness.

## 1. Introduction

Colorectal cancer (CRC), a prevalent digestive tract malignancy, is the third most frequent malignancy after lung cancer and breast cancer in the world, especially in developed countries. It is reported that CRC ranks third among cancers worldwide in terms of morbidity and mortality [1], and the incidence of CRC is also mounting in developing countries. Among patients with CRC, 5% to 10% belong to familial hereditary diseases caused by familial adenomatous polyposis, and over 90% are manifested as sporadic tumors [2]. At present, scholars have improved their cognition of the CRC and its treatments due to the improvement of modern medical levels. However, the specific indicators for early diagnosis are still not sufficient, and most CRC patients have entered the middle and advanced stages when receiving clinical diagnosis. CRC is a major hazard to human health, and in fact, the survival of CRC patients with advanced invasion and metastasis is less than 10% at 5 years [3, 4]. Therefore, it is urgent to seek appropriate markers for improvement in screening and early diagnosis of CRC.

Octamer-binding transcription factor 4 (Otc4A), a totipotent or pluripotent stem cell marker, is crucial for embryonic stem cells to retain totipotency and self-renewal. Recent studies have established the critical function of Otc4A in the gene regulatory network of stem cell development [5], and over 1,000 genes are regulated by Otc4A. Related research studies have reported Otc4A expression in many tumor cells, such as MCF-7 cell lines in human breast cancer and breast cancer tissues, pancreatic cancer Capan-2, Pan-1 cell lines, liver cancer Mahlava cell lines, gliomas, and bladder cancer tissues [6–8]. Some articles also pointed out that Otc4A overexpression can enhance stem-like properties of CRC cells, such as cell migration, invasion, and drug resistance [9]. Even yet, it is unclear exactly what part of Otc4A plays in the initiation of colorectal cancer. MicroRNAs (miRNAs) are endogenous noncoding RNAs around 18–25 nucleotides long, and specifically attach to the 3′-UTR of mRNAs to regulate an array of biological processes like gene expression, cell differentiation, proliferation, and apoptosis [10]. More than 700 miRNAs have already been identified, and it is reported that miRNAs not only are closely related to pathological processes such as tumors and inflammation [11, 12], but also have a pivotal role in predicting therapeutic strategies and targeted therapy [13]. As a tumor suppressor, MiR-7-5p prevents CRC cells from proliferating and migrating by suppressing Krüppel-like factor 4 expression [14]. However, the roles of Otc4A and miR-7-5p in CRC remain unclear. There are several subtypes of Otc4A. Otc4AA plays an important role in the early developmental stages of embryonic stem cells and in the maintenance of stem cells. The roles and functions of other variants and isoforms of Otc4A in biological systems are less well understood. In this study, we employed the Otc4AA variant as a research subject. Therefore, the effects of Otc4A and miR-7-5p on CRC were investigated in this study. This study aimed to provide reliable data support for searching for biomarkers and improving diagnosis of CRC.

## 2. Materials and Methods

### 2.1. Cell Culture

CRC cells HT29 and normal colon epithelial cells FHC were procured from the National Collection of Authenticated Cell Cultures. The purchased cells were grown in DMEM-F12 and RPMI-1640 medium supplemented with FBS (10%) and penicillin streptomycin (1%), and then CO_2_ (5%) and humidity (95%) at 37°C were set to cultivate the cells.

### 2.2. Cell Culture and Transfection

HT29 cells were digested and passed after they entered the logarithmic growth phase. The logarithmic phase cells were then gathered and diluted to 2 × 106 cells/ml. Later, a six-well plate was chosen to seed the cells. Subsequently, the transfection was conducted once the cell confluence was between 80% and 90%. Otc4A knockout plasmid (si-Otc4A) and its negative control (si-NC), miR-7-5p mimics (miR-7-5p mimics) and negative mimics (NC mimics), negative inhibitor (NC-inhibitor) and miR-7-5p inhibitor (miR-7-5p inhibitor), and pcDNA3.1-TLR4 plasmids (TLR4) were constructed by Guangzhou RiboBio Co. Ltd. (China). As per the instructions of the lipo2000 transfection kit, the vectors in each group and the liposomes were diluted with a serum-free OptiMEM culture medium. Next, the liposomes with equal volumes were mixed with the vectors in each group. After incubating at ambient temperature for 20 minutes, they were introduced to the cultured cells for another 6 h culture. Then, a complete medium was used in place of the previous medium. In the end, the cells were harvested after 48 h transfection.

### 2.3. Real-Time Fluorescence Quantitative Polymerase Chain Reaction (RT-qPCR)

Utilizing a total RNA extraction kit, the total RNA was isolated from the collected cells or tissues, and the extracted RNA was then kept at −80°C. Subsequent to the directions in the reverse transcription polymerase chain reaction (PCR) kit, cDNA was then created by reverse transcription. Following tests to ascertain the concentration and purity of the synthesized cDNA, PCR was performed under the guidelines of real-time PCR. The following was the reaction program: 95°C (1 min), 95°C (40 s), 58°C (40 s), and 72°C (45 s), with a 35-cycle, and 72°C (10 min). The data analysis technique adopted was the 2^−ΔΔCt^ method [15]. In Table 1, the primer sequences were displayed.

### 2.4. CCK-8 Method

After being digested, the logarithmic phase cells were diluted to 4 × 104 cells/mL. A further step involved seeding the cells in a 96-well plate (5000 cells per well). Additionally, each group received 6 replicate wells. The cells were cultured for 24 h, and after cell adhesion, the cells were starved by adding a serum-free medium for another 24 h. Then each well received a supplementation of fresh complete culture (90 *μ*L) and CCK-8 solution (10 *μ*L) for an additional 2 hours of culture. Ultimately, in order to detect the absorbance at 450 nm, a microplate reader was applied [16].

### 2.5. Cell Cloning Assay

The cells in the log phase were digested and passaged. The cells were counted, and the culture medium was taken to regulate cell concentration. The cells were then plated into dishes (200 cells per dish). Later, the dishes were gently shaken in a cross direction in order to disperse the cells evenly. Subsequently, static culture was conducted for 2–3 weeks in an environment of 37°C and 5% CO_2_. The colonies that had over 50 cells were counted under a microscope, and the following formula was used to calculate the colony forming efficiency: the colony forming efficiency (%) = (number of clone cells/number of seeded cells) × 100.

### 2.6. Transwell Experiments

Matrigel was removed from a −20°C environment and left overnight at 4°C. After melting the Matrigel into liquid, a serum-free medium at 4°C was introduced to the Matrigel for a 1 : 6 dilution. The upper transwell chamber insert was then filled with 100 *μ*l of Matrigel. Afterwards, the insert was placed at a temperature of 37°C for 3–5 h to solidify the Matrigel. The subsequent steps mirrored those of the migration test. Briefly speaking, the upper chamber received 100 *μ*l cells, while the lower chamber received 500 *μ*l 10% FBS medium. Subsequent to 48 hours of incubation, the cells were fixed first, then stained, and finally counted.

### 2.7. Cell Sphere-Formation Assay

Trypsin at a concentration of 0.25% was selected to lyse cells that were in a satisfactory growing state in each group. Later, the cells were collected, and they underwent two low-speed PBS washes. Further, the collected cells in each group were resuspended using the prepared stem cell culture medium, and the cell concentration was increased to 10000 cells per milliliter. Next, the resulting cells were inoculated in a 24-well culture plate (1000 cells/well), with each group occupying 3 wells. By the way, the plate was dedicated to antiadhesion. Subsequently, the plate was shaken to make the cells distribute evenly, and then the incubation was conducted at 37°C with CO_2_ (5%). After being in culture for 1–2 weeks, the cells were subjected to observation by means of a microscope. Finally, the number and the formation efficiency of cell spheres with a diameter longer than 75 *μ*m were successively obtained.

### 2.8. Dual-Luciferase Reporter Assay

When the degree of cell fusion approached 80%–90%, transfection was carried out. The cotransfection of the established NC mimics and miR-7-5p mimics into TLR4 wild-type (TLR4-WT) and mutant (TLR4-MUT) or Otc4A wild-type (Otc4A-WT) and mutant (Otc4A-MUT) dual-luciferase reporter vectors was performed subsequently. Later, the cells were collected after the transfection and 48 hours of culture. Then, a condition of room temperature was set for cell lysis for 20 min. Subsequently, the luciferase substrate was added directly after the supernatant was collected by centrifugation. Next, luciferase activity was measured using a luminescence analyzer. Additionally, relative firefly luciferase activity was determined by taking Renilla luciferase activity as an internal control.

### 2.9. Chromatin Immunoprecipitation

The culture medium was removed when the cells reached 70%–80% confluence, and the supernatant was extracted for acquiring protein. The immune-precipitated beads were vortexed and shaken for 1 min to be fully resuspended. After that, 35 *μ*l of magnetic beads were put into a 1.5 ml centrifuge tube that already contained 400 *μ*l of binding buffer. Then the beads were fully resuspended and washed. Later, the magnetic separation was performed. After the solution became clear due to magnetic bead adsorption, the supernatant was aspirated and discarded. The EP tube was removed from the magnetic separator and washed once again. Then, 400 *μ*l of binding buffer was added for the subsequent trials. The antibody binding reaction was performed as follows: on a rotary shaker at ambient temperature, the beads were first bound to the antibody for 15 minutes. Next, the beads were washed with binding buffer three times, slightly centrifuged, and rinsed three more times in the same buffer. Later, the beads and lysates were placed in a refrigerator overnight at 4°C for the last binding. After washing the beads again, magnetic separation was conducted. Subsequently, the supernatant was discarded, and 400 *μ*l washing buffer was poured into the EP tube. Again, the washing buffer was taken once more to wash the beads four times repeatedly. During the last washing process, the beads were collected into a new centrifuge tube of 1.5 ml. Elution was performed, and 25 *μ*l of 1 × SDS buffer was added. Then, the boiling water bath was conducted for 5 min. Finally, the beads were set up on a magnetic grate for magnetic separation, after which the supernatant was collected for WB detection.

### 2.10. Western Blot

Before being broken up by sonication in an ice bath, each group's cells underwent a 20-minute lysis with RIPA lysate. The protein concentration was then measured after collecting the supernatant proteins. Later, sodium dodecyl sulfate-polyacrylamide gel electrophoresis (SDS-PAGE) was performed, and the proteins were then transferred to PVDF membranes. Following that, blocking was done for 1 h at ambient temperature. After that, primary antibodies (Otc4A, p65, p-p65, TLR4, and *β*-actin) were added and the mixture was incubated at 4°C overnight. Subsequent to two rounds of washing the membranes, secondary antibodies that had been diluted and enzyme-labeled were added. After that, the membranes were incubated for another 1 h at ambient temperature. An internal control for the examination of the protein level was decided to *β*-actin.

### 2.11. Statistical Analysis

All obtained data were subjected to analysis using SPSS 26.0. Besides, *t*-test analysis was implemented between two groups, while one-way analysis of variance was conducted among multiple groups. In addition, Pearson phototropism was employed to examine how miR-7-5p, Otc4A, and TLR4 relate to one another. The test outcomes were described as mean ± standard deviation (SD), and *P* < 0.05 was regarded as the criterion for statistical significance.

## 3. Results

### 3.1. Knockdown of Otc4A Inhibits Malignant Phenotype, Stemness, and NF-*κ*B Signaling Activation in Colorectal Cancer Cells

**Figure 1:**
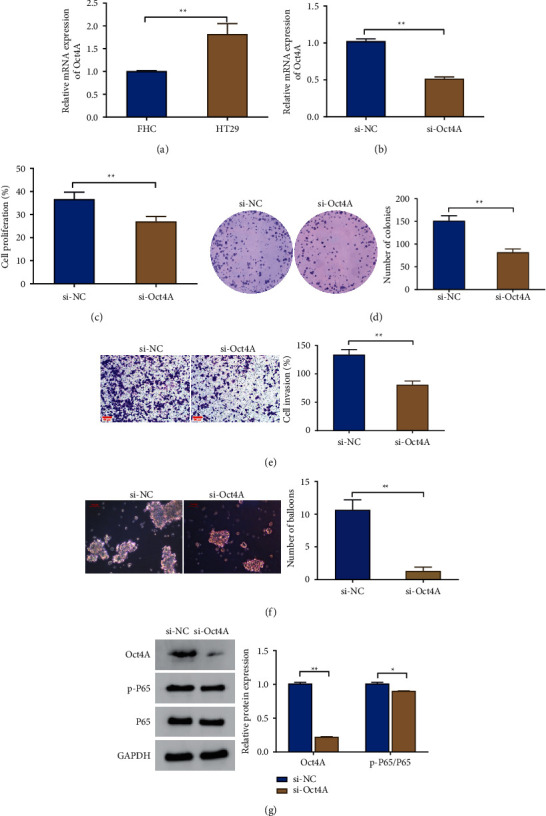
Effects of knockdown of Otc4A on the phenotype and stemness of colorectal cancer cells. qRT-PCR were used to detect Otc4A expression level in FHC and HT29 cells (a) and the si-NC and si-Otc4A groups (b); (c) CCK-8 for detecting the proliferation rate of cells; (d) cell cloning assay for the viability of cells; (e) Transwell for the invasion ability of cells; scale bar = 50 *μ*m. (f) Sphere-forming assay for observing the sphere-forming ability of cells; scale bar = 100 *μ*m. (g) Western blot for determining the expression level of Otc4A, p65, and p-p65. ^*∗*^*P* < 0.05, ^*∗∗*^*P* < 0.01. Each experiment was performed in triplicate.

To begin with, the expression and function of Otc4A in CRC cells were examined. The examination results presented that HT29 cells had much higher levels of Otc4A expression than FHC cells (Figure 1(a)). The si-Otc4A group exhibited a markedly lower level of Otc4A expression as compared to the si-NC group, indicating a successful knockdown (Figure 1(b)). After the further knockdown of Otc4A, an obvious reduction was observed in the cell proliferation rate, viability, invasion ability, and sphere-forming ability of the cells (Figures 1(c)–1(f)), and so did the expression levels of Otc4A, p-P65, and the ratio of p-P65/P65 (Figure 1(g)). Microsphere culture is one of the methods to obtain stem cells and tumor stem cells. Tumor stem cells can survive in a serum-free medium, while differentiated cells will die under such conditions. Thus, the sphere-forming ability of cells was observed by a sphere-forming assay, indicating the stemness of the tumor cells.

### 3.2. Negatively Regulatory Effect of Otc4A on miR-7-5p Expression in Colon Cancer Cells

**Figure 2:**
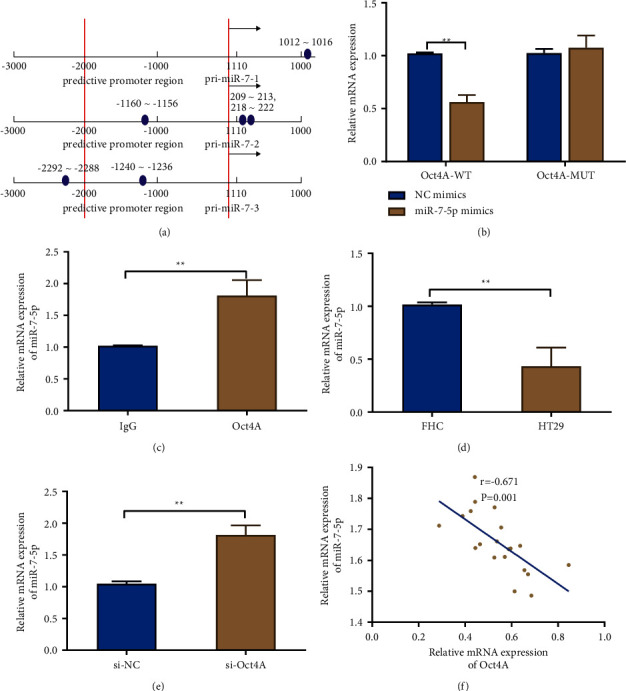
Effects of Otc4A on miR-7-5p in colorectal cancer cells. (a) The bioinformatics prediction was applied to obtain Otc4A binding sites in the miR-7-5p promoter region; (b) dual-luciferase reporter assay to verify the targeting relationship verified, ^*∗∗*^*P* < 0.01; (c) chromatin immunoprecipitation to verify the interaction between Otc4A and miR-7-5p, ^*∗∗*^*P* < 0.01; qRT-PCR to detect the expression level of miR-7-5p in FHC and HT29 cells (d) and in each group (e); ^*∗∗*^*P* < 0.01; (f) the correlation analysis for the connection between Otc4A and miR-7-5. Each experiment was performed in triplicate.

The mechanism of Otc4A affecting CRC was further explored. In the miR-7-5p promoter region, Otc4A binding sites were determined by biological information tests (Figure 2(a)). Also, the co-transfection of miR-7-5p overexpression could apparently suppress the luciferase activity of the Otc4A-WT vector but not the Otc4A-MUT vector, according to the dual-luciferase reporter assay. The above outcomes indicated the interaction between Otc4A and miR-7-5p. Additionally, chromatin immunoprecipitation findings also showed that Otc4A and miR-7-5p bind to one another (Figure 2(b) and 2(c)). Besides, HT29 cells showed considerably lower miR-7-5p expression than FHC cells (Figure 2(d)). Furthermore, miR-7-5p expression was significantly elevated in the cells when Otc4A was knocked down, and an inverse correlation between Otc4A and miR-7-5p was discovered (Figures 2(e) and 2(f)).

### 3.3. TLR4 Serves as a Downstream mRNA of miR-7-5p

**Figure 3:**
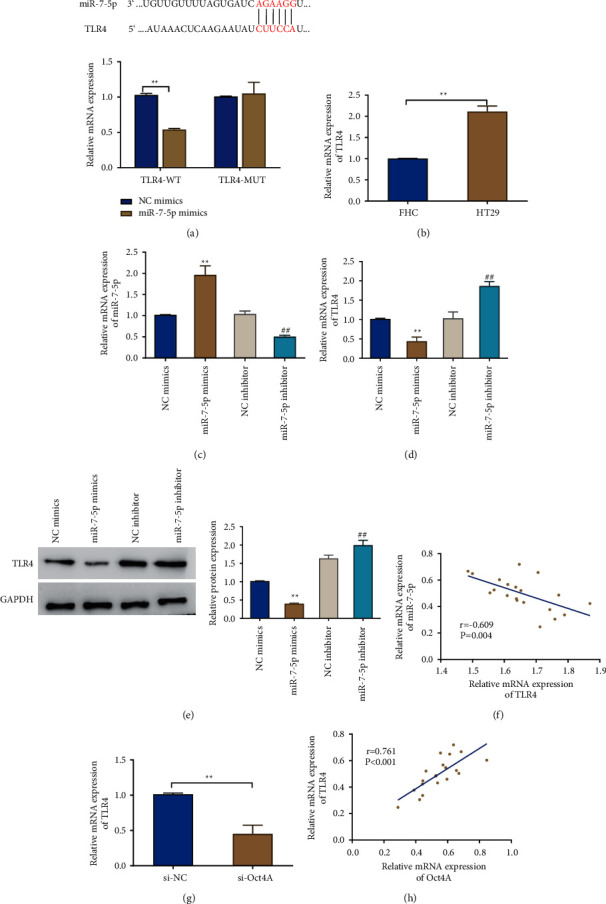
Effects of miR-7-5p on TLR4 expression in colon cancer. (a) Database was utilized to predict the binding sites of miR-7-5p and TLR4; dual-luciferase reporter assay to verify the targeting relationship between miR-7-5p and TLR4; (b-d) qRT-PCR to check TLR4 expression level in FHC and HT29 cells (b) as well as miR-7-5p (c) and TLR4 (d) expression level in each group of cells; (e) Western blot to detect the protein level of TLR4 in cells; (f) correlation analysis for miR-7-5p and TLR4 expression; (g) qRT-PCR to observe the expression level of TLR4 in the si-NC and si-Otc4A groups; and (h) Correlation analysis for the association between Otc4A and TLR4 expression. ^*∗*^*P* < 0.05, ^*∗∗*^*P* < 0.01, ^##^*P* < 0.01. Each experiment was performed in triplicate.

The downstream targets of miR-7-5p were further investigated. The Targetscan database (Fig. https://www.targetscan.org/vert_72) predicted as well as discovered the sites binding miR-7-5p and TLR4. Besides, the dual-luciferase reporter assay also indicated that overexpressing miR-7-5p considerably inhibited the luciferase activity of the TLR4-WT vector while having no obvious impact on the TLR4-MUT vector (Figure 3(a)). The above findings proved the targeting link between miR-7-5p and TLR4. Additionally, HT29 cells had significantly higher TLR4 expression than FHC cells did (Figure 3(b)). When miR-7-5p was overexpressed, its expression level went up, while the knockdown of miR-7-5p significantly declined the expression level in cells (Figure 3(c)). This result suggested a successful cell transfection. The levels of TLR4 mRNA and protein expression were correspondingly decreased or increased upon overexpression or knockdown of miR-7-5p in cells (Figures 3(d) and 3(f)). The correlation analysis also further showed that TLR4 was negatively correlated with miR-7-5p (Figure 3(f)). When compared to the si-NC group, the TLR4 expression level in the cells was noticeably lower in the si-Otc4A group, and Otc4A was positively correlated with TLR4 (Figures 3(g) and 3(h)).

### 3.4. Otc4A Regulates the Malignant Progression of HT29 Cells through miR-7-5p/TLR4 Axis

**Figure 4:**
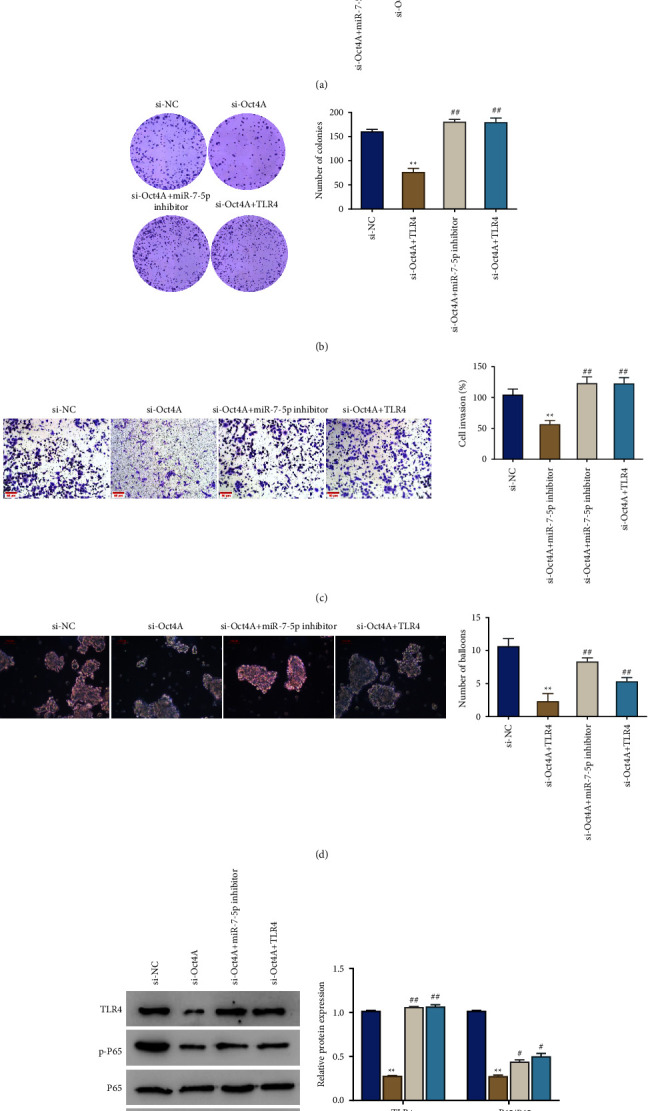
Roles of Otc4A, miR-7-5p, and TLR4 in colorectal cancer. (a) CCK-8 was adopted to detect the proliferation rate of cells; (b) cell cloning assay to confirm the viability of cells; (c) Transwell assay to detect the invasion ability of cells; scale bar = 50 *μ*m. (d) Sphere-forming assay to observe the sphere-forming ability of cells; scale bar = 100 *μ*m. (e) Western blot to detect the expression level of TLR4, p65, and p-p65 in cells. ^*∗∗*^*P* < 0.01*vs*. the si-NC group; ^#^*P* < 0.05, ^##^*P* < 0.01*vs*. the si-Otc4A group. Each experiment was performed in triplicate.

In this part, the roles of Otc4A, miR-7-5p, and TLR4 in CRC were further researched. We acquired the following results: firstly, different from the si-NC group, the si-Otc4A group exhibited a significant decrease in the proliferation rate, viability, invasion ability, sphere-forming ability, number of spheroids, and the expression level of TLR4 and p-p65, as well as the ratio of p-p65/p65 of the cells. However, the si-Otc4A + miR-7-5p inhibitor and si-Otc4A + TLR4 groups showed a remarkable increase in the proliferation rate, viability, invasion ability, sphere-forming ability, number of sphere-forming ability, TLR4 and p-p65 expression levels, and the ratio of p-p65/p65 of the cells, compared with the si-Otc4A group (Figure 4).

## 4. Discussion

CRC is the third most common malignancy after lung and breast carcinoma worldwide (especially in developed countries), and its incidence is increasing in developing countries [17]. As one of the heterogeneous cell populations with various characteristics, CRC arises with cancer cells of the colon, rectum, and appendix. Additionally, genes and the microenvironment are important factors in CRC pathogenesis [18]. In spite of the effectiveness of surgical and chemotherapy treatments, the prognosis allows for no optimism considering that many CRC patients suffer tumor recurrence or metastasis after surgery. One of the major clinical challenges of CRC is advanced diagnosis. Numerous gene mutations, dysregulated signaling networks, or an evident genetic variation among individuals all contribute to the complex pathogenesis of CRC. Therefore, molecular biological studies of biomarkers have important clinical implications.

Otc4A is included in the POU family of transcription factors. As a totipotent or pluripotent stem cell marker, Otc4A is critical for embryonic stem cells to retain totipotency and self-renewal. Otc4A is expressed in many different types of tumors, and its expression is associated with the tumorigenicity of pluripotent cells [19]. However, relevant studies on the expression of Otc4A in CRC tissues and its role in CRC are rare at home and abroad. Wang et al. [20] claimed that the positive expression of Otc4A could be a warning sign for distant recurrence and poor prognosis of rectal cancer in patients undergoing chemoradiotherapy before surgery. Studies have shown that Otc4A protein overexpression plays a role in the development of CRC in the development of colon cancer, and Otc4A is a key transcription factor for maintaining the survival of colon cancer tumor transcription factor critical for the survival of stem cell-like cells [21]. NF-*κ*B falls into the category of a nuclear factor that binds to the gene regulatory region encoded by the immunoglobulin *k* light chain of activated B cells, and the abnormal activation of NF-*κ*B could enhance cancer cell proliferation by binding to the cyclin D1 promoter [22]. Furthermore, the NF-*κ*B signaling pathway promotes angiogenesis by regulating proangiogenic factors, vascular endothelial growth factor, and the proinflammatory cytokine IL-8 in cancer cells. A number of malignancies are impacted by abnormal NF-*κ*B expressions [23]. NF-*κ*B consists of five proteins, which are bound to I*κ*B*α* in the cytoplasm at rest to form a complex. When I*κ*B*α* is activated to form p-I*κ*B*α*, I*κ*B*α* is ubiquitinated and degraded to release bound NF-*κ*B p65, which is further phosphorylated to form p-NF-*κ*B p65, enhancing its localization signal into the nucleus to promote downstream gene expression. P65 is the most widely distributed heterodimer. Therefore, the NF-*κ*B signaling pathway can be expressed by P65. In this study, knockdown of Otc4A inhibited the proliferation, migration, sphere formation, and NF-*κ*B signaling activation of HT29 cells. The above findings in this study suggested that Otc4A was a cancer-promoting transcription factor, and with it, the NF-*κ*B signaling pathway could be activated and CRC cell proliferation could be induced.

Numerous studies have revealed microRNAs (miRNAs) as novel and useful biomarkers that can be employed in the early diagnosis, treatment, and prognostic estimation of CRC [24]. Recent studies on the biological relationship between miR-7 and tumors have gradually increased. Some studies have discovered that a number of malignant tumors, such as CRC, lung carcinoma, and breast carcinoma, exhibit abnormal expression of miR-7 that implicates the proliferation, differentiation, and apoptosis of tumor cells; and miR-7 also affects tumor growth, invasion, and metastasis [25]. Besides, MiR-7 can target p21-activated kinase 1, epidermal growth factor receptor, and insulin receptor substrate 1 to influence biological processes like proliferation, migration, apoptosis, and cell cycle of tumor cells in CRC by targeting [26]. Giles et al. [27] also came to the conclusion that miR-7-5p had the ability to inactivate the NF-*κ*B pathway to suppress the growth and metastasis of melanoma, which suggests that miR-7-5p has the potential to be a treatment target for melanoma. Till now, however, there have been few studies on the connection between miR-7-5p and CRC. The membrane receptor TLR4 is closely related to intracellular inflammation and immune response. To be specific, when TLR4 is activated, MyD88 is urged to recruit the TIR domain of TLR4; then, the degradation of I*κ*B*α* and the activation of NF-*κ*B are induced; the transcription of proinflammatory factor genes such as TNF-*α*, IL-6, and IL-1*β* is enhanced; and the synthesis and release of TNF-*α*, L-6, and IL-1*β* are correspondingly increased; then the increase in the contents of TNF-*α*, IL-6, and IL-1*β* further strengthens the activation of NF-*κ*B, promotes the nuclear transfer of NF-*κ*B, and then aggravates immune inflammation [28]. In this paper, Otc4A was found to be a negative regulator of miR-7-5p, and TLR4 was found to be a downstream mRNA of miR-7-5p. Moreover, through the miR-7-5p/TLR4 axis, Otc4A regulated the proliferation, invasion, cell stemness, as well as NF-*κ*B signaling of HT29 cells. The above results suggested that Otc4A is significant in promoting the malignant biological behavior of HT29 cells, and its mechanism may be achieved by preventing the miR-7-5p/TLR4 axis from activating the NF-*κ*B signaling pathway.

In this study, it was found that the transcription factor Otc4A can regulate the level of TLR4 by inhibiting the expression of miR-7-5p and then promoting the proliferation and invasion of CRC cell HT29 and enhancing cell stemness. However, there are still some limitations to this study: First, we only conducted in vitro experiments, lacking evidence of in vivo experiments. We can collect patient samples and conduct further experiments in animals. Second, we did not further detect biomarkers of other stem cells. Finally, experiments were conducted on only one CRC cell line, and more cells could be used in the future.

## 5. Conclusion

Taken together, the cancer-promoting transcription factor Otc4A activates the NF-*κ*B signaling pathway by reducing the regulatory effect of miR-7-5p on TLR4 level and then enhancing the proliferation, invasion, and cell stemness of CRC cells HT29. This study lays a solid experimental foundation for exploring biomarkers in the diagnosis and treatment of CRC.

## Figures and Tables

**Table 1:** Primer sequences for qRT-PCR.

Genes	Primer sequences
Otc4A	F 5′-GAACATGTGTAAGCTGCGGCC-3′
	R 5′-CCCTTCTGGCGCCGGTTAC-3′

miR-7-5p	F 5′-TGCGCTCAGCAAACATTTATTG-3′
	R 5′-CCAGTGCAGGGTCCGAGGTATT-3′

TLR4	F 5′-CTGGGTGAGAAAGCTGGTAA-3′
	R 5′-AGCCTTCCTGGATGATGTTGG-3′

U6	F 5′-CTCGCTTCGGCAGCACA-3′
	R 5′-AACGCTTCACGAATTTGCGT-3′

GAPDH	F 5′-GTGAACCATGAGAAGTATG-3′
	R 5′-CGGCCATCACGCCACAGTTTC-3′

## Data Availability

The datasets used and/or analyzed during the current study are available from the corresponding author on reasonable request.
